# A prospective study of physician pre-hospital anaesthesia in trauma patients: oesophageal intubation, gross airway contamination and the ‘quick look’ airway assessment

**DOI:** 10.1186/1471-2253-13-21

**Published:** 2013-09-11

**Authors:** David J Lockey, Pascale Avery, Timothy Harris, Gareth E Davies, Hans Morten Lossius

**Affiliations:** 1 London’s Air Ambulance, Department of Pre-hospital Care, Royal London Hospital, London E1 1BB, UK; 2 Department of Research and Development, The Norwegian Air Ambulance Foundation, Holterveien 24, PO Box 94, N-1441 Drøbak, Norway; 3 Field of Pre-hospital Critical Care, Network of Medical Sciences, University of Stavanger, Kjell Arholmsgate 41, NO-4036 Stavanger, Norway

**Keywords:** Tracheal intubation, Airway, Pre-hospital, Anaesthesia

## Abstract

**Background:**

In trauma patients intubated in a physician-led pre-hospital trauma service we prospectively examined the rate of misplaced tracheal tubes, the presence and nature of gross airway contamination, and the value of ‘quick look’ airway assessment to identify patients with subsequent difficult laryngoscopy.

**Methods:**

Patients requiring pre-hospital intubation in a 16 month period were included. Intubation success rate, misplaced tracheal tube rate, Cormack and Lehane grade, and the presence and nature of gross airway contamination were recorded at laryngoscopy. Tube placement was verified with carbon dioxide detection and chest x-ray. After visual assessment physicians stated whether laryngoscopy was expected to be a straightforward or ‘difficult’. The assessment was compared to subsequent laryngoscopy grade.

**Results:**

400 patients had attempted intubation and 399 were successfully intubated. 42 were in cardiac arrest and intubated without drugs. There were no oesophageal or misplaced tracheal tubes. Gross airway contamination was reported in 177 of 400 patients (44%), of which ¾ was from the upper airway. Unconscious patients had higher contamination rates (57%) than conscious patients (34%) (p ≤ 0.0001). As a test of difficult intubation, the ‘quick look’ generated sensitivity 0.597 and specificity 0.763 (PPV and NPV were 0.336 and 0.904 respectively).

**Conclusion:**

This study suggests that when physicians perform pre-hospital anaesthesia they have high intubation success rates and the use of ETCO2 monitoring reduces or eliminates undetected misplaced tracheal tubes. We found high rates of airway contamination; mostly blood from the upper airway. The ‘quick look’ airway assessment had some utility but is unreliable in isolation.

## Background

Published reports suggest trauma patients requiring emergency intubation present more difficulties than patients presenting for elective anaesthesia [1]. In addition, airway management in the pre-hospital phase of care is usually carried out in suboptimal conditions and considered to be more difficult [1,2]. Although pre-hospital advanced airway management is a well described, complex intervention, the characteristics of trauma patients presenting to pre-hospital doctors for advanced airway management and the frequency of difficulties encountered during their management has not been extensively reported [2]. This information might be used to help quantify the risks of pre-hospital anaesthesia and airway management and target training. This is particularly relevant in many European countries where physician led pre-hospital care is common [3-5] and in the UK where a national report supported physician pre-hospital anaesthesia for trauma patients with airway compromise [6].

Formal airway assessment prior to intubation is difficult to achieve in seriously injured trauma patients and is rarely carried out in our pre-hospital system. However detection of a difficult airway could influence advanced airway management techniques and improve successful intubation rate. Although carbon dioxide detection devices do not improve intubation skills or success rates they do prevent unrecognised oesophageal intubation. Undetected oesophageal intubation is catastrophic, but remains prevalent [7] despite widespread availability of end tidal carbon dioxide monitoring devices. Furthermore, the rate of gross airway contamination in trauma patients is not often reported and the source (from above or below) has implications particularly when use of alternative airway management devices is considered.

This study was carried out to provide more information on the rate of oesophageal or misplaced tracheal tube placement and the rate and nature of visible airway contamination in a physician-manned service that provides pre-hospital anaesthesia for trauma patients. We also wanted to establish whether a rapid visual assessment of the airway by the attending physician was of any value in predicting subsequent difficult view at laryngoscopy.

## Methods

### Study ethics

Local ethics committee (East London and The City Research Ethics Committee 2) were approached but formal ethical approval was not required because no treatment changes were made and additional data was only collected about standard practice. The project was therefore registered as a service evaluation.

### Study environment

The system in which this study was based is described in accordance with the recently published ‘fixed system variables’ for uniform reporting of data from advanced airway management in the field [8]. The study was carried out in London UK in an urban physician-led pre-hospital trauma service serving a daytime population of around 10 million in an area of approximately 5,000 square kilometres. A doctor – paramedic team is delivered by helicopter in the daytime and by fast response car at night. Flight paramedics work in the ambulance control room and use dispatch criteria and call interrogation to target patients with severe trauma. The doctor – paramedic team are always dispatched in addition to a standard land ambulance response. The service attends an average of 5-6 trauma patients per day. Doctors are experienced emergency physicians or anaesthetists with in-hospital anaesthetic experience and pre-hospital training. Emergency physicians are expected to have a minimum of six months full time training in anaesthesia. Anaesthetists have completed a minimum of three years full time anaesthesia training. Both are also subject to pre-hospital anaesthesia training in the initial pre-hospital training period. All doctors carrying out advanced airway intubations in the study period participated in the study. Flight paramedics have specific training to equip them as members of the pre-hospital anaesthesia team. Pre-hospital anaesthesia is carried out in line with UK recommendations on pre-hospital anaesthesia [9] and local standard operating procedures. Most patients are anaesthetised for the following indications: actual or impending airway compromise, ventilatory failure, unconsciousness or cerebral agitation. A simple and highly reproducible anaesthetic technique is used and the on-scene doctor has a limited number of treatment choices to make. The technique is well rehearsed and each member of the team has clearly defined roles. A pre-induction checklist is carried out before administration of induction drugs. In the study period anaesthesia was induced with etomidate and suxamethonium was used for initial muscle relaxation. Sedation was then maintained with morphine and midazolam and muscle relaxation with pancuronium. Intubation is carried out routinely with a standard Macintosh size 4 blade with the standard use of an intubating catheter (Cook Medical, Frova airway intubating catheter™). Cricoid pressure is routinely applied and laryngeal manipulation carried out when requested by the operator to improve laryngoscopic view. Equipment for failed intubation includes a supraglottic airway and equipment for surgical cricothyroidotomy. Patients are wherever possible anaesthetised on an ambulance trolley with 360 degree access to optimise laryngoscopic view. A mechanical ventilator is available to ventilate patients after intubation (Drager™ Oxylog 2000). Time intervals routinely recorded include: time of emergency call, time of activation, time of arrival on scene, induction time, time departing scene and time of arrival in hospital.

### Study variables

The pre-hospital doctor recorded data prospectively shortly after each mission. The same patient cohort was used for the study of a number of variables in airway management including the effect of cricoid pressure [10] and internal comparisons of operator intubation success [11]. For this study, in addition to standard database information on incident type, patient and interventions, specific information was recorded on whether difficult intubation was predicted, intubation success and the Cormack and Lehane [12] view at initial laryngoscopy. Management of failed intubation was also recorded. After the decision to intubate was made, the doctor was required to verbalise to the flight paramedic and document whether, on the basis of rapid external visual assessment (with no fixed criteria), the patient would be difficult or easy to intubate. This assessment was then linked to intubation success and laryngoscopic view. Cormack and Lehane views grade I and II were classified as ‘easy’ and grade III and IV as ‘difficult’. Successful tube placement was established on scene by colourimetric and continuous quantitative CO2 monitoring and, in the emergency department, with continuous CO2 monitoring. Tracheal tube position was also routinely assessed on initial chest X- Ray after arrival in the emergency department. Presence and nature of visible airway contamination (vomit, blood or both) was recorded at direct laryngoscopy. After every mission that included pre-hospital general anaesthesia, pre-hospital doctors were required to complete an anonymised airway data form in addition to standard database information.

### Statistical analysis

Pearson chi-square values were determined for categorical data using IBM SPSS Statistics 20 software. Confidence intervals were calculated for proportion of successful intubations using Microsoft Excel.

## Results

Data was recorded prospectively over a sixteen-month period (1^st^ January 2007 to 31^st^ May 2008). Thirty-two doctors attended 481 patients who required tracheal intubation. 347 (72%) were male and 134 (28%) were female. 441 (91%) were victims of blunt trauma and 40 (9%) victims of penetrating trauma. Seventy-nine were intubated without drugs by attending paramedics prior to physician arrival. Two had immediate surgical airways performed by physicians without attempted laryngoscopy because patient entrapment made laryngoscopy difficult. Four hundred had attempted intubation. Forty-two were in cardiac arrest and had uncomplicated intubation without the use of drugs. Of the remaining 358 patients, 357 were intubated orally after induction and one (0.3%) had a surgical airway after failed intubation. The intubation success rate was 99.75% (399/400) [95% CI 0.993-0.999]. Of the 400 patients who had laryngoscopy the Cormack and Lehane grades were (at initial view) Grade 1: 254 patients (64%), Grade 2: 79 patients (20%), Grade 3: 48 patients (12%), Grade 4: 20 patients (5%). The initial view was not recorded in two patients (0.5%).

Among the intubated patients there were no oesophageal or misplaced tracheal tubes. In the study population gross airway contamination was reported in 177 out of 400 patients (44.3%). Of the 177 patients 138 (78%) had contamination with blood, and 26 (14.7%) had contamination with vomit. Ten patients (5.6%) had contamination with both. In three patients (1.7%) the airway was contaminated with teeth or brain matter. Where GCS immediately prior to intubation was recorded, patients with a GCS 3-8 (unconscious) were more likely to have a contaminated airway (57%), compared to patients with a GCS 9-15 (conscious) (34%) (p ≤ 0.0001,). We are aware that it has been suggested that regurgitation of vomit into the airway may be associated with gastric inflation as a result of bag-valve mask ventilation. Unfortunately we were unable to establish whether a high proportion of the patients who had airway contamination with vomit had bag-valve-mask ventilation prior to laryngoscopy.

Prior to laryngoscopy doctors were asked to briefly look at the patient and predict whether they thought that laryngoscopy would be difficult. At laryngoscopy an ‘easy’ view was defined as Cormack and Lehane grade I or II and ‘difficult’ as grade III or IV. Results of this visual assessment are shown in Table 1. When doctors predicted that the grade would be difficult it was much more likely to be so: 34% were grade III or IV compared with 9.6% where the visual assessment predicted a good laryngoscopy grade (p ≤ 0.0001, Fisher exact test). As a test of difficult intubation, the ‘quick look’ generated sensitivity 0.597 (0.47-0.76) and specificity 0.763 (0.71-0.81). The positive and negative predictive values were 0.336 (0.25-0.43) and 0.904 (0.86-0.94) respectively, with a likelihood ratio of 2.52. (Values have 95% confidence interval).

**Table 1:** The ‘quick look’ airway assessment - predicted difficulty and laryngoscopy grade

		**Difficult**	**Easy**	**Total**	
Prediction from the ‘quick look’ assessment		119 (30%)	281 (70%)	n	%
				400	100
Grade of laryngoscopy at first look	I & II	79 (20%)	254 (64%)	333	83
	III & IV	40 (10%)	27 (7%)	67	17

## Discussion

Pre-hospital advanced airway management is a complex intervention and many factors may influence patient outcome. This study specifically reported physician success rates and the high intubation success rate reported in this study compares well with the intubation success rates of pre-hospital physicians in similar studies [13-16]. A recent meta-analysis examining intubation success rates of EMS providers demonstrated overall intubation success rates of 92.7% for all EMS providers, 95.5% for paramedics using drugs to assist intubation, and 99.1% for physicians. The available data on pre-hospital physician intubation success is limited and was based on 2,536 attempted intubations [13]. Our reported intubation success rates are also similar to those reported in emergency department intubation. Success rates of 99.7% have been reported in emergency departments in both the US [17] and the UK [18]. The physicians performing intubation in this study were a mix of emergency physicians and anaesthesiologists. A recent paper from Germany commented on the different success rates between ‘proficient’ and ‘expert’ performers (defined by the number of intubations performed per year) [18] – ‘expert’ performers had a higher intubation success rate and a lower incidence of difficult laryngoscopy. It is likely that this study contained a mixture of ‘proficient’ and ‘expert’ performers when the same definitions are used.

The Cormack and Lehane laryngoscopy grading is a commonly used system to describe laryngeal view at direct laryngoscopy [12]. The rates of ‘difficult’ laryngoscopy (grades III and IV) reported in this study (17%) are comparable to rates previously reported in pre-hospital studies (13-19%), but much greater than rates described in emergency department patients (8.5%), and patients presenting for elective anaesthesia (6.1%) [19-22]. Possible reasons for this difference include different operating conditions, operator experience and patient casemix. These results emphasise the importance of carefully structured pre-hospital anaesthesia and intubation protocols to minimize risk. Structured guidelines for advanced pre-hospital airway management have been published in the UK [9], Scandinavia [23] and the US [24] and have much in common. Since the intubation success rate was very high in this group of physician ‘expert’ and ‘proficient’ performers it might be concluded that a poor Cormack and Lehane grade at laryngoscopy is not necessarily associated with failed intubation. However this study does not record intubation difficulty only ultimate success.

There were no oesophageal intubations or misplaced tracheal tubes in this study. This may be due to a ‘team approach’ to induction where misplaced tubes are actively sought by the doctor and paramedic and tube confirmation confirmed by a combination of direct vision at laryngoscopy, qualitative capnometry, quantitative capnography and auscultation. Despite the ready availability of low cost carbon dioxide detection devices for the rapid detection of oesophageal intubation, several studies have reported significant unrecognised oesophageal intubation rates. One recent study from a well-developed EMS system described a 31% failed intubation rate with the use of muscle relaxants, and a 12% unrecognised oesophageal intubation rate [25]. The introduction of capnography has been clearly demonstrated to reduce the rate of unrecognised oesophageal intubation. A study in a US regional EMS evaluating misplaced intubation rates with and without CO2 monitoring observed 23% and 0% misplaced tube rates before and after the introduction of CO2 monitoring [26]. The potentially catastrophic complication of unrecognised oesophageal intubation can be prevented by the mandatory use of capnometry. This should be considered an essential item for advanced airway management both in and out of hospital [9,27-29]. There were 42 patients in this study intubated in cardiac arrest. In this group of patients detection of carbon dioxide is likely to be less effective at confirming tracheal tube position.

Despite the fact that the techniques used in emergency airway management are highly influenced by the risk of aspiration the method of detection of significant airway contamination is poorly defined and the clinical significance of aspiration of gastric contents is largely unclear. Presence of gross airway contamination may not accurately reflect the risk of aspiration and morbidity, but is highly likely to reflect an increased risk of aspiration in trauma patients. The high gross airway contamination rate reported in this study after inspection at laryngoscopy (44.3%) confirms gross contamination is common in this patient group. The higher reported rate of contamination in unconscious patients is expected and likely to be due to an inability to respond appropriately to airway soiling. Data published in a previous smaller study reported similar contamination rates (34%) [30]. A US study compared incidence of aspiration of gastric contents in patients intubated in the pre-hospital setting and the emergency department [31]. Aspiration rates of 50% and 22% were reported respectively; further supporting higher airway contamination rates occurring in the pre-hospital patient population. Of note in our study, contamination was predominately from upper airway blood (78%) rather than gastric contents (15%), comparable to smaller study findings (83% and 17% respectively) [30]. These figures have implications for pre-hospital airway management, where definitive airway control with a tracheal tube is indicated but might not be possible in the early phases of care. The use of supraglottic airway devices provides a degree of protection from contamination from above. Reliable airway protection from aspiration of blood has been clearly demonstrated with the use of supraglottic devices in elective surgery. In one study of 200 patients having elective tonsillectomy 98% of patients had no or minimal contamination of the LMA, 2% had contamination in the cup of the LMA, but there was no observed soiling of the tube [32]. These results suggest that use of supraglottic airways in the pre-hospital patient population may provide good protection from airway contamination from the upper airway in the majority of patients. It has also been suggested that bag-valve-mask ventilation may increase risk of gastric inflation and aspiration of stomach contents but no reliable data exists to support this hypothesis [33]. This study did not attempt to investigate the relationship between airway management prior to laryngoscopy and the presence of gross contamination. Early use of supraglottic devices may help prevent this theoretical risk, but the true implications of airway contamination in pre-hospital advanced airway management should be further explored.

There is limited published data on the prediction of intubation difficulty in the pre-hospital population. Airway assessment scores have been used successfully in the emergency department population to stratify the risk of intubation difficulty [34]. The ‘LEMON’ assessment described by Walls et al [35] uses five components to assess likely difficult intubation (Table 2). They consist of an external assessment looking for features associated with difficult intubation (‘quick look’) and evaluation of the ‘3-3-2’ rule (assessment of maximum mouth opening by assessing distance between upper and lower incisors, hyoid-mental and thyroid to mouth distance which should be greater than 3,3 and 2 finger breaths respectively). The Mallampati score [36] (which involves airway assessment in the sitting position), airway obstruction and assessment of neck mobility complete the assessment. Validity and practicality of some elements of this assessment technique have been questioned in the emergency department patient. Mallampati score was shown to be both difficult to assess, and a poor predictor of intubation grade [17]. The practical use of an assessment that requires a sitting patient, placing fingers in a patient’s mouth and testing the mobility of the neck are all questionable in the pre-hospital trauma patient. The potential use of the ‘quick look’ test for prediction of difficult intubation has been previously described in a physician based EMS in Germany [20]. There was some correlation between physician’s prediction and laryngoscopic findings. The positive and negative predictive values of this ‘quick look’ test of difficult intubation produced comparable results (0.46, 0.95 respectively) to the results in this study. The ‘quick look’ assessment in our study appears to have some utility in predicting difficult laryngoscopy in pre-hospital trauma patients but is unreliable in isolation. Where a ‘quick look’ predicted a difficult airway it was much more likely to be difficult. It may be that there is a genuine link between external assessment and laryngoscopic view, but there is also the possibility that the physicians may have been influenced by a subjective expectation of negative outcome.

**Table 2:** One method of assessment of the emergency airway

**The ‘LEMON’**	**Airway assessment method (adapted from Reed MJ 2005**^**33**^**)**
L	Look externally for characteristics known to cause difficult laryngoscopy, intubation or ventilation.
E	Evaluate the 3-3-2 rule (incisor distance 3 finger breadths, hyoid-mental distance 3 finger breadths, thyroid – mouth distance 2 fonger breadths)
M	Mallampati Score (≥ 3)
O	Obstruction (conditions such as trauma, epiglottitis, peritonsillar abcess
N	Neck mobility (limited neck mobility)

### Limitations

In this study findings should be interpreted with caution due to low rates of some reported complications. The ‘quick look’ and Cormack and Lehane grading system relied on self-reporting by the doctor-paramedic team of laryngoscopic views and success rates and may be subject to reporting bias. Also there is a possibility that unrecognised clinical, environmental, operational or educational factors might have influenced the results.

The significance of gross upper airway contamination was not investigated and may not correlate with clinical aspiration syndromes. Details on the number of attempts at intubation, time taken for intubation to be achieved and physiological derangements were not recorded in this study. The intubation data has to be interpreted with caution in the absence of this information.

Lastly, since this study was performed in a physician–led pre-hospital trauma service the results may not be applicable in EMS systems where airway management is carried out by non-physicians.

## Conclusion

This study reports on pre-hospital anaesthesia carried out in trauma patients by physicians. This study reports a high rate of difficult laryngoscopy, a high intubation success rate, no misplaced tracheal tubes, but a high rate of airway contamination in this patient group. A ‘quick look’ airway assessment had some utility but is unreliable in isolation.

## Competing interests

The authors declare that they have no competing interests.

## Authors’ contributions

DL and TH planned the study and collected the data. DL and PA wrote the manuscript. DL, PA, TH, GD and HML read, critically appraised and contributed to manuscript revision. All authors read approved the final manuscript.

## Pre-publication history

The pre-publication history for this paper can be accessed here:

http://www.biomedcentral.com/1471-2253/13/21/prepub
